# Concerns over functional experiments, interpretation, and required controls

**DOI:** 10.1172/JCI157369

**Published:** 2022-03-01

**Authors:** Eddie C.Y. Wang, Ceri A. Fielding, Richard J. Stanton

**Affiliations:** Division of Infection and Immunity, School of Medicine, Cardiff University, Cardiff, United Kingdom.

**Keywords:** COVID-19, Immunology, NK cells

## To the Editor:

Hsieh et al. (1) provide comprehensive data correlating NK phenotypes with SARS-CoV-2 clearance. However, the functional experiments provided to support their mechanistic conclusions are far from definitive and are missing the correct comparators required for appropriate interpretation.

In Figure 3 and Supplemental Figure 5, the authors claim that CD155 and nectin-4 are upregulated by SARS-CoV-2. Nucleocapsid expression in partially infected cultures was used to differentiate infected from uninfected cells, yet uninfected cells in this system respond to interferons and cytokines released by infected cells (2); CD155 and nectin-4 levels must be compared to levels in mock-infected cells. Furthermore, the magnitude of the changes is small. Without showing absolute changes (e.g., flow cytometry plots), it is impossible to determine whether effects are biologically meaningful. Interpretation of immunofluorescence staining of infected lung tissue is also problematic. End-stage COVID patients have severe inflammation, which upregulates CD155. Demonstrating that this effect is specific to live cells productively infected with SARS-CoV-2 requires a much more robust quantitative comparison of uninfected and infected cells. Additionally, viruses frequently retain NK ligands intracellularly (3–5); thus, the presented histological staining is inappropriate, as it cannot differentiate intracellular from cell-surface staining.

The authors use spike pseudotyped lentivirus to claim that SARS-CoV-2 upregulates CD155, resulting in decreased NK killing in animal models. However, pseudovirus is only appropriate to measure viral entry. By the time of these assays, spike protein has been lost, and no other SARS-CoV-2 genes are present. These assays investigate the effect of lentiviral vector–mediated RFP expression. No conclusions about SARS-CoV-2 can be drawn.

The authors claim that SARS-CoV-2–mediated upregulation of CD155 enables the virus to evade NK control, yet in other viruses, downregulation of CD155 inhibits NK cells, since DNAM1 is dominantly activating (3), counter to their claims.

In Figure 4A, the absence of mock-infected controls in their TIGIT-Fc NK experiments means no conclusions can be drawn about SARS-CoV-2. It is simply demonstrating TIGIT’s involvement in NK function.

The comparison of two donors (Figure 4D) does not allow conclusions about DNAM function; these donors demonstrate differences in DNAM1; however, they will contain numerous differences in other NK ligands. Without additional controls (e.g., blocking DNAM1), it is impossible to conclude anything about DNAM1’s involvement.

In summary, we believe that conclusions relating to the alteration of the cell surface by SARS-CoV-2, the ability of NK cells to control SARS-CoV-2, and the involvement of the DNAM1/TIGIT pathway in this process are not supported by many of the experiments presented in this paper, which should be interpreted with more caution.

## Version 1 03/01/2022

electronic publication
